# Simulation-based training of junior doctors in handling critically ill patients facilitates the transition to clinical practice: an interview study

**DOI:** 10.1186/s12909-018-1447-0

**Published:** 2019-01-08

**Authors:** Søren Marker, Marlene Mohr, Doris Østergaard

**Affiliations:** 1 Copenhagen Academy for Medical Education and Simulation, Herlev Hospital, Capital Region of Denmark and Copenhagen University, Herlev Ringvej 75, 2730 Herlev, Copenhagen, Denmark; 2 Department of Intensive Care 4131, Copenhagen University Hospital Rigshospitalet, Inge Lehmanns Vej 5, 2100 Copenhagen, Denmark

**Keywords:** Simulation-based learning, Emergency situations, Context adaptation, Knowledge transfer, Algorithms

## Abstract

**Background:**

Junior doctors lack confidence and competence in handling the critically ill patient including diagnostic skills, decision-making and team working with other health care professionals. Simulation-based training on managing emergency situations can have substantial effects on satisfaction and learning. However, there are indications of problems when applying learned skills to practice. Our aim was to identify first-year doctors’ perceptions, reflections and experiences on transfer of skills to a clinical setting after simulation-based training in handling critically ill patients.

**Methods:**

We used a qualitative approach and conducted semi-structured telephone interviews with a sample of twenty first-year doctors six months after a 4-day simulation-based training course in handling critically ill patients. Interviews were transcribed verbatim. A content-analysis approach was used to analyse the data.

**Results:**

The following main themes were identified from the interviews: preparedness for clinical practice, organisational readiness, use of algorithms, communication, teamwork, situational awareness and decision making. The doctors gave several examples of simulation-based training increasing their preparedness for clinical practice and handling the critically ill patient. The usefulness of algorithms and the appreciation of non-technical skills were highlighted and found to be helpful in managing clinical difficulties. Concern was expressed related to staff willingness and preparedness in using these tools.

**Conclusions:**

Overall, the simulation-based training seemed to facilitate the transition from being a medical student to become a junior doctor. The doctors experienced an ability to transfer the use of algorithms and non-technical skills trained in the simulated environment to the clinical environment. However, the application of these skills was more difficult if these skills were unfamiliar to the surrounding clinical staff.

**Trial registration:**

Not applicable.

**Electronic supplementary material:**

The online version of this article (10.1186/s12909-018-1447-0) contains supplementary material, which is available to authorized users.

## Background

The transition from being a medical student, with limited responsibilities and a high level of supervision, to become a newly qualified doctor, with medical responsibility for individual patients and less supervision, is difficult and stressful [1–4]. Especially, being able to identify the deteriorating patient, to make decisions and prepare a plan for the patient is a challenge [5]. Junior doctors experience high levels anxiety related to feeling responsible for making decisions about patient care and working alone with less supervision [2]. Accordingly, confidence in history taking, but less confidence in diagnostic skills and decision-making is reported. Junior doctors experience that finding the right diagnosis and initiating a treatment plan was different from the student perspective, where decisions seemed more clear cut [2].

One explanation for this might be the focus on the medical expert role and on stable patients in medical schools [5]. Thereto, typically doctors have responsibility for several patients at a time, which implies being able to prioritize and collaborate in a team of healthcare professionals. Junior doctors find these aspects difficult [5].

The educational methods used in medical schools are primarily based on lectures, which do not include the possibility of training and applying these skills [6]. The importance of interactive training methods e.g. simulation-based training (SBT) as a methodology for learning is growing rapidly in healthcare [7, 8]. In post-graduate medical education, Denmark, in line with several countries, has adapted the CanMed 7 roles [9]. These roles encompass the non-technical skills (NTS), such as situation awareness, decision-making, communication, teamwork and leadership. Advantages of SBT may include improved self-confidence, less anxiety, and improved feeling of being proficient [10]. Participants are generally satisfied with SBT, and typically learning outcome is reported as high in post-test [11, 12]. Although participants apply what they learn, over time problems with maintaining what has been learned may arise [13].

A recent review demonstrates that transfer of knowledge and skills might be impaired during acute events [14], but little is known about which personal and contextual factors facilitate or impair transfer of learning from simulation to clinical settings [15–17]. A deeper understanding of these aspects would be helpful when designing SBT [4].

We speculated whether a SBT course aiming at combining medical expertise skills and NTS could help first-year doctors transfer these skills to the clinical setting.

Hence the aim of this study was to identify first-year doctors’ perceptions, reactions and reflections on transfer of skills after SBT in handling critically ill patients.

## Methods

We conducted an explorative interview study in the Capital Region in Denmark. Participants received oral and written information about the study and gave verbal informed consent.

### Context

The National Board of Health describes the overall goal of the clinical training of first-year doctors. In short, the aim of the first year is to learn how to function as a doctor and work together with other health professionals.

All first-year doctors in the capital region participate in a 4-day mandatory SBT course during the first two months of clinical practice (2 days in the first month and 2 days in the second month) after the graduation from the university (please find the detailed course program and organisational information in the Additional file 1: Appendix 1). The overall aim of the course is to prepare first-year doctors for their clinical work: to handle the critically ill patient, to understand the importance of NTS for the medical task management and to apply these skills in a team. This mandatory course was developed by a group of doctors from anaesthesia, internal medicine and surgical specialties in collaboration with a team of pedagogical experts from the simulation centre. The course is conducted 10 times a year, which means that the course has been evaluated and changed over time. The instructors are experienced doctors certified in in SBT and in conducting feedback/ debriefings. The course takes place in a realistic hospital environment at the Copenhagen Academy for Medical Education and Simulation, Copenhagen University Hospital, Herlev.

The theoretical content includes: cardiac arrest (day 1), the critically ill patient in the medical and surgical ward (day 2 and 3) and handling transportation of the critically ill patient (day 4).

The learning objectives represent a combination of theoretical and practical skills aiming at enhancing the doctors´ diagnostic and medical expertise skills as well as their NTS. The doctors are along the course trained in the use of concepts and algorithms, such as the ABCDE (Airway, Breathing, Circulation, Disability and Examination) approach [18] to critically ill patients; the SBAR (Situation, Background, Assessment, Recommendation) algorithm [19] for handover of patient information; and the ACCEPT (Assessment, Control, Communication, Evaluation, Preparation/Packaging and Transport) algorithm [20] for plan and preparation of patient transport (the algorithms are further elaborated on in the Additional file 1: Appendix 1).

The course consists of short lectures, workshops and SBT using advanced patient simulators. The SBT scenarios include simulated patients with acute medical conditions, which the first-year doctor has to handle. The learning objectives in each scenario encompass both medical expertise skills and NTS. The SBT scenarios, which lasts around 20 min are followed by 25 min’ feedback (day 1 and 4) and debriefing using clips from video recordings (day 2 and 3). The debriefing is structured in 3 phases: a description -, analysis - and application phase [21].

The typical team size for SBT is 5 doctors role-playing a first-year doctor, a senior doctor and one or two nurses. One or two doctors observe the team, focusing on specific learning objectives for subsequent discussion.

### Study design and study population

A purposive sample of first-year doctors, who had participated in a course, were recruited; 34 doctors participated in the training. Thirty-three participants (all these unfamiliar with the interviewer) received an invitation to participate in individual interviews. One course participant was not invited as he had been working in the simulation centre during his medical education. The first author, who is not involved in the conduct of the courses, recruited the participants by e-mail, followed by a telephone call to non-responders. All interviews were carried out exactly 6 months after the course to ensure 6 months of hospital clinical practice.

SM conducted semi-structured telephone interviews using an interview guide (available in the Additional file 1: Appendix 1). The guide is modified from an interview guide developed for a similar type of study [17]. Each interview was recorded using a digital audio voice recording device and transcribed verbatim by the first author. We used systematic text condensing to extract categories as recommended by Malterud [22]. In Step 1, all interview recordings were listened to by SM; two authors (SM and DO) read the initial 5 transcripts in their entirety to get an overall impression of the data and create preliminary themes. In Step 2, the authors identified and coded text fragments (using primary codes). In Step 3, systematic abstraction of the text fragments was done by creating a condensate (maintaining reference to appropriate interviews). Authentic illustrative quotations were also identified for each main theme. In Step 4, we conceptualized data by writing an analytic text for each main theme, making sure that the synthesized results still reflected the validity and wholeness of their original context.

## Results

The study comprised 20 first-year doctors with 6 months’ clinical experience at the time of the interview. Saturation of interview data was reached around the tenth interview. However, all interviews scheduled at time of data saturation were performed. Eleven female doctors (55%) and nine male doctors (45%) participated. Interviews lasted 15–25 min, except for one interview, which were cut short after 5 min. No interviewees needed additional interview time or a second interview.

Table 1 shows the main themes identified in the interviews and the narrative illustrations supporting the themes related to reactions and reflection on transfer of skills to the clinical setting. The interview statements, described in detail below, clustered around the following themes: preparedness for clinical practice, the use of algorithms and NTS.

**Table 1:** Main themes, subthemes and quotations related to the first-year doctors’ reactions and reflections on transfer of learning from the course to the clinical setting

Main themes	Subthemes	Quotations
Preparedness for clinical practice	*Handling critically ill patients,* e.g. *sepsis*	[INT6]’… When you are there with your first critically ill patients you really need to know where to start and that is what you learn from this course’ ’…At first you maybe panicked a little - but then you calmly started to work…’
		[INT14]’… You have to go and see a patient you don’t know who is really not feeling well and then you have this systematic approach…’
		[INT12]’…I think the patient transfer part of the course totally changes your way of working, totally. And you feel more confident. Previously, I have been thinking “I don’t even know this patient” and emphatically browsed the patient chart while heading for the scanner’
	*Maintaining an overview*	[INT7] “…You maintain an overview and remain calm in the situation”…often it is the nurse who loses grip of the situation’
	*Feedback, debriefing*	[INT11] ‘After this course, I’ve had use of feedback/debriefing afterwards, in the sense that we have evaluated after cardiac arrest situations, for example’
Organisational readiness	*The organisation is not ready to receive*	[INT12] ‘This thing with closed loops and to think aloud, I think it was really good and I have really tried to use it afterwards. Some of the older colleagues think it gets a little too weird and start to give too many inputs, but the younger nurses have really liked it and responded positively’
Algorithms – useful tools that work in clinical praxis	*ABCDE approach for assessment*	[INT5]’…Patient with dyspnoea… and then you do and ABCDE-assessment and because you do it systematically you find out it is actually a… which you wouldn’t have done just as fast otherwise’
	*ACCEPT approach for transfer of critically ill patients’*	[INT4]’…When transferring a patient from the ER to the ward I used this ACCEPT approach prior to the transfer. And it worked really well. I felt more secure about having the equipment with me, but also it actually worked as a kind of debriefing for the nurses who had been in the resuscitation room in the ER when he arrived’
	*SBAR format for structured communication regarding a patient*	[INT9] ‘I have used SBAR a lot and I have implemented it, so I don’t really think about it anymore’ ’…Need a basic structure to keep track of who you are talking to, what the situation is about and stuff like that’
Communication	*Capability of talking aloud*	[INT6]’…And talking aloud about the things and doing a systematic evaluation’
	*Giving orders*	[INT2]’…To keep on saying what you are doing and to say I need this and that. You can feel the positive feedback afterwards, because people need it’
	*The necessity of a clear recipient*	[INT15] In cardiac arrest. ‘And you must think about speaking directly to people, to make sure they hear and understand what you say - and you hear what they want to say’
	*Structured communication*	See also: *algorithms – SBAR* [INT9] I have used SBAR a lot and I have implemented it, so I don’t really think about it anymore’
Teamwork	*Understanding the role of other team members*	[INT4]’…Easier to cooperate with the nurses in the acute situations afterwards’ ‘I remember to ask: how far are you?’
		[INT6] ‘Due to this training I can see that sometimes other professions contribute with valuable input, so you have to think aloud and remain focused’
		[INT2] ‘You are more aware of the information your colleagues need in these situations. It really is teamwork and it is especially in these situations you get tested and become most aware of whether it works’
Leadership	*Take the role as a leader*	[INT11]’… After the course I wanted to be team leader in a cardiac arrest situation and I would not have liked that before’
Situational awareness	*Plan and prepare*	[INT19] Transfer of trauma patients to the CAT scan:’…and then suddenly a bell rang and I said, okay, I need the emergency kit, − I need to consider what potentially might happen, which medication I would potentially need, and then the whole process was better.’ ’… And I would actually not have done this prior to this course’
Decision making	*Being able to make decisions*	[INT18] ‘In real life I would, in some of the situations in the simulation room, have asked someone, call my attending physician or someone else and I think it is an advantage that you feel confident in making some decisions - that changes your mind set compared to beforehand’

*INT* Interview number, *ABCDE* Airway, Breathing, Circulation, Disability, Exposure, *ACCEPT* Assessment, Control, Communication, Evaluation, Preparation/Packaging and Transport, *SBAR* Situation, Background, Analysis, Recommendation, *CAT* Computer Assisted Tomography

### Preparedness for clinical practice

The doctors gave several examples of increased preparedness for the handling of critically ill patients following attendance of the course. Dealing with critically ill patients were seen as the most challenging and anxiety-provoking elements when starting clinical work as a doctor and the simulation scenarios addressed these situations.

Their anxiety was diminished by using a systematic approach to the critically ill patient. This was found to facilitate rational thought and actions in time-critical situations and thereby boost the self-confidence. The training was accordingly reported to facilitate the transition from the theoretical approach at the university to the highly practical clinical situations.

### Algorithms

Algorithms and Mnemonics were in general found to be useful and implemented in the clinical settings. The interviewees felt that this provided them with tools to fall back on when losing focus. The ABCDE, ACCEPT and SBAR-algorithms were widely perceived to be the key learning goals of the course. The ABCDE approach allowed for a systematic approach to every critically ill patient, contributing with the structure and overview that would otherwise have been missed. The ACCEPT approach were reported to be of great relevance to the first-year doctors, who often accompany critically ill patients to diagnostic interventions (e.g. CAT-scans) or transfer between hospitals. The ACCEPT approach was found to increase the awareness of potential complications during transportation and allow for adequate measures to be taken in advance. The SBAR tool were to some extent found useful, however in general reported not to be followed rigorously by staff members. Concerns were raised about most hospital staff not being ready to facilitate the use of these tools. Especially, it was stressed that many senior doctors were novices in the use of algorithms.

### Non-technical skills

The use of NTS, in clinical practice was found to be helpful in meeting the medical expertise challenges. The examples of increased focus on situational awareness were reported alongside the use of the ABCDE and ACCEPT algorithms. Increased focuses on communication during and after treating critically ill patients were reported, highlighting the SBT course as the reason for this. Especially the need for clear recipients of communication when initiating different medical treatments was emphasized. The implementation of principles such as closed loops communication was by several interviewees said to be impeded by nursing staff not responding in similar fashion.

One participant felt a genuine development in self-confidence after having to make decisions that would have normally been made by a more senior colleague. Another participant highlighted that being ‘forced’ to act was a rewarding way of professional development and that this development was encouraged by feeling safe in the team. Some mentioned they felt safer knowing it was not a real patient situation and that, therefore, they could make mistakes without consequences. Another participant mentioned that feeling the pressure to act creates the realism, and in real life one often feels even more pressure, emphasizing that SBT provides the opportunity of psychological preparation.

In Table 2 the main themes related to the evaluation on course content, format and structure is seen. Overall, the comments were positive about both the content of the course focusing on the use of structured tool to facilitate the initial treatment of the critically ill patient and the NTS. The doctors high-lighted the importance of a safe learning environment and appreciated the combination of the different methods.

**Table 2:** Main findings related to the first-year doctors evaluation of the course content, format and structure

Course aspect	Main findings
Pre-course preparation	▪ Course preparation is beneficial. ▪ A low level of updated theoretical medical knowledge on the subject impairs focus on the desired learning regarding non-technical skills
Course content	▪ Standardised tools/algorithms are what is remembered – they allow you to regain focus when lost in emergency situations ▪ The individual gain and motivation depends on the actual current work-related context (department) of the doctor. Handling critically ill patients daily probably increases an individual’s motivation for course participation ▪ Updating and refreshing existing knowledge and capabilities are needed
Level of scenario difficulty and scenario focus	▪ Level of difficulty needs to be balanced ▪ More direct feedback related to the simulated patient treatments is wanted ▪ Keeping focus in the course on the relevant issues in the sudden transition from ‘theory to praxis’ experienced by the foundation year doctors and addressing the critical situations that may be feared accordingly is important
Theoretical lectures vs. simulation training	▪ The balance between theory and praxis and the time for reflection generates optimal outcome from the course
Playing other professions in SBT	▪ There is a pronounced learning potential in playing different professions in simulation, e.g. realising the necessity of good communication strategies and adjusting the expectations and attitudes to ‘real life’ teamwork. ▪ However, the most rewarding is playing the role of your own profession.
SBT with other health professions.	▪ Training with other health professions (e.g. nurses) might be valuable – they are the ones you are surrounded by in a real-life situation with a critically ill patient.
Realism in simulation	▪ Scenario realism is important for maximizing the gain from the course – you forget it is not a ‘real life’ situation ▪ This realism depends on the attitude of the participants – if one refuses to ‘play’, realism drops heavily ▪ The scenario complexity has to be limited to allow focus on relevant learning goals and preventing chaos impairing learning.
Safe learning environment	▪ Feeling safe without the risk of embarrassment in front of colleagues is important for the learning outcome of the course. ▪ Individuals feel ‘safer’ when everyone participates; rotating roles for each simulated scenario, within the same group, and by knowing it is not a real patient. ▪ A moderate feeling of pressure to act in the simulated context is necessary and contributes with potential mental preparation for ‘real-life’ situations.
Recapitulation	▪ There was considerable satisfaction with the course content and structure ▪ Simulation is an effective and popular learning method

*SBT* Simulation-based training

The doctors expressed that training with other health professionals e.g. nurses might be beneficial. Nurses are part of the team and the ones to collaborate with in clinical situations emphasizing that nurses should also train the same algorithms and NTS. It might be that transfer of skills would be enhanced if both professions were trained together. Hence it was suggested to develop multi-professional SBT courses in handling critically ill patients.

## Discussion

The interviews reveal first-year doctors´ perceptions and reactions after a simulation-based training course and suggest that newly graduated doctors are able to transfer skills acquired in a simulated setting to a clinical setting. The course was a bridge from the highly theoretical content of medical schools to the clinical work as a health professional strongly relying on human interactions and managing cooperative tasks.

The content and the interactivity of the SBT courses seem to have facilitated the first-year doctors’ confidence in their ability to handle critically ill patients. This is in agreement with the findings in a questionnaire survey of medical students´ perception and confidence after a simulation based exercise [23] and suggestions from Tallentire and colleagues [4]. The value of algorithms and structured tools, such as the ABCDE approach; the ACCEPT algorithm for planning patient transportation; and SBAR for handing over patient information, were found to support situational awareness and decision-making in their clinical work providing tools to fall back on when loosing focus. In line with our results, gaining practice in application of ABCDE has elsewhere been cited as the primary benefit after SBT [24]. After a course for medical students, SBAR was the most cited benefit followed by NTS and ABCDE [23].

The benefit of structured tools and algorithms correlates to the Dreyfus & Dreyfus 1980 [25] categorisation of the five steps in human learning processes. The novice is more in need of guidelines and structure. In our study the participants expressed that not all senior doctors acknowledged the use of algorithms. This is in agreement with a previous study of Advance Life Support (ALS) course participants’ difficulty in returning from a course to the clinical environment where not all have been trained [17].

The training of first-year doctors in NTS was found useful in relation to subsequent clinical practice. This is in agreement with the findings of Watmough et al. [24]. The SBT was focused on providing opportunities for the doctors to make decisions, based on the theory, that you cannot learn how to make decisions without making decisions. All scenarios resemble the clinical situations first-year doctors are likely to encounter in the beginning of their careers. The course had particular focus on the many facets/roles of being a doctor, e.g. leader, collaborator and communicator, aiming at making the doctor realise the importance of these roles for the optimal handling of the patient. The NTS are part of specialist training programmes in anaesthesiology and surgery and it seems valuable to introduce first-year doctors to these skills [26].

Our results are in agreement with the findings of Watmough et al., who engaged final year medical students in unexpected simulation scenarios while they were in clinical training [24]. The training was evaluated immediately after the course (questionnaire) and when the students had begun working as first-year trainee (interviews). The results indicate that participants felt the program helped them as junior doctors in terms of dealing with emergency situations such as a deteriorating patient [24].

The doctors expressed overall satisfaction with the content, the combination of the methods used and the safe learning atmosphere. Our training programme was in line with the recommendations for running teaching programmes for newly qualified doctors [27].

The course engaged the participants in the SBT and the feedback/debriefing helped them to reflect on their actions and set new individual learning objectives. This is in line with theories of adult learning. It was mentioned that more specific feedback on elements of lacking competence might have been useful. This is in agreement with the recent literature indicating that it is also important to identify gaps and peaks of performance [28]. Feedback given in a more direct manner is frequently used in the clinical setting and after courses such as the ALS course [17, 26]. We used a more reflective debriefing structure, where the instructor guides the learner by asking questions, as reflection has been identified as central in learning [7, 21, 28].

The potential advantages of SBT are numerous. It is possible to train in a safe environment and adjust the scenarios according to the learner’s needs. For instance, if the learner needs more time for decision-making, the deterioration of the patient can be slowed down. In the simulated setting, education can be the primary priority, whereas in the clinical setting, patient safety always comes first. The drawbacks of SBT are the high costs of the technology and the costs of taking the doctors away from their clinical work.

### Discussion of the method used

One of the limitations of this study is the size of the study population. However, saturation of interview data was seen from the 10th interview. Susceptibility to participation bias cannot be ruled out given the study design with a limited study population of volunteering course participants. The reporting of important aspects in this study was secured by referring to the ‘Consolidated criteria for reporting qualitative studies’ (COREQ): 32-item checklist described by Tong et al. 2007 [29].

All participants came from the same educational region and there might be a risk that this was not representative at a national or international level. However, the observed transfer of learning from the simulation setting to the clinical setting can be of interest to medical educators worldwide.

The strength of the study is the standardisation of the course and the scenarios as well as the training of the instructors. Interviewing the young doctors 6 months after the course allowed reflection on the usability of SBT-acquired skills in the clinical setting. Evaluating transfer of skills following SBT at this particular time in the doctors ‘ongoing education’ furthermore minimises the influence of other optional SBT courses, as these are typically attended after the first clinical foundation year.

Our study contributes to the understanding of how to use SBT to optimize learning [15] and highlight factors of importance in transfer of skills to clinical settings. We found an overall wish to include other health professions e.g. nurses in SBT The literature supports this tendency towards inclusion of the relevant professions in training [30].

This study has added knowledge about transfer from the simulated to the clinical environment. We realised how important it is to inform the senior staff about the learning objectives in the course in order to facilitate transfer and maintain the first-year doctors´ use of algorithms and guidelines. The first-year doctor might play an important role in the implementation of new knowledge and skills in the organisation. The study indicates a need to include focus on algorithms and NTS in pre-graduate medical education.

## Conclusions

This study suggests that SBT in handling critically ill patients is useful in facilitating the transition from being a medical student to the subsequent clinical work of first-year doctors. The doctors felt better prepared and experienced that they were able to transfer the algorithms and NTS from the simulated environment to the clinical environment. An important factor in this transition is the motivation, facilitated by the realism of the simulated scenarios and insight in the potential benefit of the skills trained. Finally, this study underlines the challenge in applying new approaches in clinical practice if these are unfamiliar to surrounding staff.

## Additional file

Additional file 1: Appendix 1. Includes the following content: Semi-structured telephone interview guide, Description of algorithms, Course program, Course setting, Course expenses/costs (PDF 426 kb)

## Abbreviations

ABCDE (algorithm): Airway, Breathing, Circulation, Disability, Exposure
ACCEPT (algorithm): Assessment, Control, Communication, Evaluation, Preparation/Packaging and Transport
ALS: Advanced Life Support
CanMed: Canadian medical association
COREQ: Consolidated criteria for reporting qualitative studies
NTS: Non-technical skills
SBAR (algorithm): Situation, background, analysis, recommendations
SBT: Simulation-based training
