# Supplementary material for: Simulation-based training of junior doctors in handling critically ill patients facilitates the transition to clinical practice: an interview study
Source: BMC Med Educ. 2019 Jan 8;19:11. doi: 10.1186/s12909-018-1447-0 (PMC6323692; doi:10.1186/s12909-018-1447-0)
Supplement: Supplementary file 1 — Appendix 1. Includes the following content: Semi-structured telephone interview guide, Description of algorithms, Course program, Course setting, Course expenses/costs (PDF 426 kb) [file 12909_2018_1447_MOESM1_ESM.pdf]

<u>**ADDITIONAL FILE 1**</u>**:**

**ELECTRONIC SUPPLEMENTARY APPENDIX**

This appendix has been provided by the authors to give readers additional information about their work.

Supplement to: Marker S, Mohr M, Østergaard D. Simulation-based training of junior doctors in handling critically ill patients facilitates the transition to clinical practice: An interview study

**Electronic Supplementary Appendix for:**

**Simulation-based training of junior doctors in handling critically ill patients facilitates the transition to clinical practice: An interview study**


Semi-structured telephone interview guide ........ 3
Algorithms ........ 4
ACCEPT ........ 4
ABCDE ........ 5
(I)SBAR ........ 6
Course program ........ 7
Day 1 ........ 7
Day 2 ........ 8
Day 3 ........ 9
Day 4 ........ 10
Course setting ........ 12
Course expenses / costs for the 4-day course ........ 13
References ........ 14

**Semi-structured telephone interview guide**

As I wrote in the e-mail I sent you, I would like to hear your thoughts, reflections… *whatever*… comes into your mind when you think about the 4-day mandatory course you attended at the beginning of your clinical foundation year training… I want you to know that I am aware that it has been a while since the course, so please take the time you need… And please let me know if I say something you do not understand or if you need any other clarification.

1) Firstly, I would like you to think back to the course and tell me what comes into your mind regarding your immediate reactions to the course.

> *This could be regarding the course … content, format, the tests or the teachers, the participants … anything … that comes into your mind when you think back?*

2) During the time since you attended the course, which clinical situations or experiences do you recall where your behaviour, thoughts, reactions … had some kind of relation to the course

> *If possible, can you describe two kinds of such situation: one that was a positive experience and one that was a negative experience?*

> *For each situation – What was the context? What was your role? Who else was involved in the situation? What did you do? How did that work out? What are your thoughts and reflections on that situation?*

Thank you so much for taking your time to talk to me.

**Algorithms**

**ACCEPT**[1]

| | | |
|---|---|---|
| **A** | **Assessment** | *History*<br>*Primary survey (ABCDE)*<br>Problem, Action, Effect, Next |
| **C** | **Control** | Team leader<br>Team members<br>Task |
| **C** | **Communication** | (I)SBAR<br>Contact information on relevant persons / departments<br>What is needed? |
| **E** | **Evaluation** | Is the plan agreed with receiver?<br>Triage: When + mode + who (competencies) |
| **P** | **Preparation[1] and Packaging[2]** | **M**edical: Patient secured and accessible?<br>**I**nstrumental: Estimated transport time?<br>**N**ursing: Care during transport?<br>**T**ransport: Equipment → Gurney/patient bed → ambulance |
| **T** | **Transportation** | Pre-transport check<br>Revaluations (using ABCDE)<br>Anticipate potential problems and have a have back-up-plan(s)<br>Handover (records, laboratory, evaluation, audit, return?) of the patient (and patient responsibility) |

*(I)SBAR: Identification, Situation, Background, Assessment, Recommendation*
*ABCDE: Airways, Breathing, Circulation, Disability, Exposure*

**ABCDE**[2]

| | | |
|---|---|---|
| **A** | **Airway** | Patent airway / threatened airway? Cervical spine?<br><br>Oxygen |
| **B** | **Breathing** | RF, SAT, inspection, palpation, percussion of thorax,<br><br>Auscultation (lungs), cyanosis, ABG. |
| **C** | **Circulation** | CRT, skin, HR, BP, ECG-monitoring, auscultation (heart), 2 x IV-access, fluids, lab. tests, hourly urinary output |
| **D** | **Disability** | AVPU / GCS score, pupils, Focal neurological deficiencies, Blood glucose level |
| **E** | **Exposure** | Look top-to-toe, temperature, avoid hypothermia |

*RF: Respiratory frequency/rate*
*SAT: Oxygen saturation*
*ABG: Arterial blood gas*
*CRT: Capillary Refill Time*
*HR: Heart Rate*
*BP: Blood pressure*
*ECG: Electrocardiography*
*IV: Intravenous*
*AVPU: Alert-Verbal-Pain-Unresponsive*
*GCS: Glasgow Coma Scale*

**(I)SBAR**[3]

| | | |
|---|---|---|
| **<u>I</u>** | **Identification** | Who is calling / answering<br><br>What is needed |
| **<u>S</u>** | **Situation** | Who is the patient<br><br>Problem |
| **<u>B</u>** | **Background** | Action<br><br>Effect<br><br>ABCDE status<br><br>Medical history (brief) |
| **<u>A</u>** | **Assessment** | Problem |
| **<u>R</u>** | **Recommendation** | What is needed |

*ABCDE: Airways, Breathing, Circulation, Disability, Exposure*

**Course program**

**Day 1**

<table>
<tr><td rowspan="1">08.30 – 09.00</td><td colspan="6">Welcome, introduction and presentation</td></tr>
<tr><td>09.00 – 09.30</td><td colspan="6">ALS lecture</td></tr>
<tr><td>09.30 – 09.45</td><td colspan="6">Coffee Break</td></tr>
<tr><td rowspan="3">09.45 – 11.00</td><td colspan="6">Skill stations</td></tr>
<tr><td colspan="3">Group 1</td><td colspan="3">Group 2</td></tr>
<tr><td colspan="3">1. BLS and safe ext. defibrillation<br>2. Airways</td><td colspan="3">1. Airways<br>2. BLS and safe ext. defibrillation</td></tr>
<tr><td rowspan="3">11.00 – 12.15</td><td colspan="6">ALS scenario practice and ABCDE practice</td></tr>
<tr><td colspan="2">Group 1</td><td colspan="2">Group 2</td><td colspan="2">Group 3</td></tr>
<tr><td colspan="2">Patient scenario practice and evaluation</td><td colspan="2">Patient scenario practice and evaluation</td><td colspan="2">Patient scenario practice and evaluation</td></tr>
<tr><td>12.15 – 12.45</td><td colspan="6">Lunch break</td></tr>
<tr><td rowspan="3">12.45 – 14.15</td><td colspan="6">ALS scenario practice</td></tr>
<tr><td colspan="2">Group 1</td><td colspan="2">Group 2</td><td colspan="2">Group 3</td></tr>
<tr><td colspan="2">Patient scenario practice and evaluation</td><td colspan="2">Patient scenario practice and evaluation</td><td colspan="2">Patient scenario practice and evaluation</td></tr>
<tr><td>14.15 – 14.30</td><td colspan="6">Coffee Break</td></tr>
<tr><td rowspan="3">14.30 – 15.45</td><td colspan="6">ALS scenario practice</td></tr>
<tr><td colspan="2">Group 1</td><td colspan="2">Group 2</td><td colspan="2">Group 3</td></tr>
<tr><td colspan="2">Patient scenario practice and evaluation</td><td colspan="2">Patient scenario practice and evaluation</td><td colspan="2">Patient scenario practice and evaluation</td></tr>
<tr><td>15.45 – 16.00</td><td colspan="6">Concluding remarks and evaluation</td></tr>
</table>

*ALS: Advanced Life Support (Advanced cardiac arrest handling)*
*BLS: Basic Life Support (Basic cardiopulmonary resuscitation)*
*ABCDE: Airways, Breathing, Circulation, Disability, Exposure*

**Day 2**

| | | | | |
|---|---|---|---|---|
| 08.30 – 08.45 | Welcome<br>Introduction to day 2 | | | |
| 08.45 – 09.30 | Team work and (I)SBAR algorithm | | | |
| 09.30 – 09.45 | Coffee break | | | |
| 09.45 – 10.15 | "The Critically ill patient" | | | |
| 10.15 – 10.30 | Briefing and instruction to the simulation room and mannequin | | | |
| | | | | |
| | Group 1 | Group 2 | Group 3 | Group 4 |
| 10.30 – 10.55 | Simulation 1 | Simulation 1 | CRM exercise | CRM exercise |
| 11.00 – 11.25 | Debriefing 1 | Debriefing 1 | Simulation 1 | Simulation 1 |
| 11.30 – 11.55 | CRM exercise | CRM exercise | Debriefing 1 | Debriefing 1 |
| 12.00 – 12.30 | Lunch break | | | |
| | | | | |
| | Group 1 | Group 2 | Group 3 | Group 4 |
| 12.30 – 12.55 | Simulation 2 | Simulation 2 | Case discussions (intoxications) | Case discussions (intoxications) |
| 13.00 – 13.25 | Debriefing 2 | Debriefing 2 | Simulation 2 | Simulation 2 |
| 13.30 – 13.55 | Case discussions (intoxications) | Case discussions (intoxications) | Debriefing 2 | Debriefing 2 |
| 13.55 – 14.15 | Coffee break | | | |
| | | | | |
| | Group 1 | Group 2 | Group 3 | Group 4 |
| 14.15 – 14.40 | Simulation 3 | Simulation 3 | Intro. to home assignment | Intro. to home assignment |

| | | | | |
|---|---|---|---|---|
| 14.45 – 15.10 | Debriefing 3 | Debriefing 3 | Simulation 3 | Simulation 3 |
| 15.15 – 15.40 | Intro. to home assignment | Intro. to home assignment | Debriefing 3 | Debriefing 3 |
| 15.45 – 16.00 | Evaluation and presentation of day 3 | | | |

*(I)SBAR: Identification, Situation, Background, Assessment, Recommendation*
*CRM: Crew Resource Management*[4]

**Day 3**

| | | | | |
|---|---|---|---|---|
| 08.30 – 08.45 | Welcome<br>Introduction to day 3 | | | |
| 08.45 – 09.15 | Follow-up on home assignment | | | |
| 09.15 – 09.30 | Coffee break | | | |
| | | | | |
| | Group 1 | Group 2 | Group 3 | Group 4 |
| 09.30 – 10.10 | Setting priorities | Setting priorities | Setting priorities | Setting priorities |
| | | | | |
| | Group 1 | Group 2 | Group 3 | Group 4 |
| 10.10 – 10.35 | Simulation 1 | Simulation 1 | “Acute Abdomen” | “Acute Abdomen” |
| 10.40 – 11.05 | Debriefing 1 | Debriefing 1 | Simulation 1 | Simulation 1 |
| 11.10 – 11.35 | “Acute Abdomen” | “Acute Abdomen” | Debriefing 1 | Debriefing 1 |
| 11.35 – 12.15 | Lunch break | | | |
| | | | | |
| | Group 1 | Group 2 | Group 3 | Group 4 |
| 12.15 – 12.40 | Simulation 2 | Simulation 2 | Case discussions | Case discussions |
| 12.45 – 13.10 | Debriefing 2 | Debriefing 2 | Simulation 2 | Simulation 2 |

| | | | | |
|---|---|---|---|---|
| 13.15 – 13.40 | Case discussions | Case discussions | Debriefing 2 | Debriefing 2 |
| 13.40 – 14.00 | Coffee break | | | |
| | | | | |
| | Group 1 | Group 2 | Group 3 | Group 4 |
| 14.00 – 14.25 | Simulation 3 | Simulation 3 | Leadership & team work | Leadership & team work |
| 14.30 – 14.55 | Debriefing 3 | Debriefing 3 | Simulation 3 | Simulation 3 |
| 15.00 – 15.25 | Leadership & team work | Leadership & team work | Debriefing 3 | Debriefing 3 |
| 15.25 – 16.00 | Evaluation<br>Presentation of day 4 | | | |

**Day 4**

| | | | | |
|---|---|---|---|---|
| 08.30 – 08.45 | Introduction to day 4 | | | |
| 08.45 – 09.30 | ACCEPT algorithm | | | |
| 09.30 – 09.45 | Coffee break | | | |
| | Group 1 + 2 | | Group 3 + 4 | |
| 09.45 – 10.25 | Paramedic/EMS providers education. Equipment/personnel/patient | | Paramedic/EMS providers education. Equipment/personnel/patient | |
| | Group 1 | Group 2 | Group 3 | Group 4 |
| 10.25 – 11.25 | Simulation 1 | Simulation 2 | Simulation 3 | Simulation 4 |
| 11.25 – 12.10 | Lunch break | | | |

| | | | | |
|---|---|---|---|---|
| 12.10 – 13.10 | Simulation 2 | Simulation 3 | Simulation 4 | Simulation 1 |
| 13.10 – 14.10 | Simulation 3 | Simulation 4 | Simulation 1 | Simulation 2 |
| 14.10 – 14.25 | Coffee break | | | |
| 14.25 – 15.25 | Simulation 4 | Simulation 1 | Simulation 2 | Simulation 3 |
| 15.25 – 15.45 | Special circumstances / reality | | | |
| 15.45 – 16.00 | Evaluation and concluding remarks | | | |

*ACCEPT: Assessment, Control, Communication, Evaluation, Preparation & Packaging, Transportation*
*EMS: Emergency Medical Services*

**Course setting**

**CAMES - Copenhagen Academy for Medical Education and Simulation**

CAMES consolidates the Capital Region of Denmark's research, development and training activities in the fields of medical and surgical simulation.

*CAMES activities at Herlev Hospital (Herlev Ringvej 75, 2730 Herlev, Greater Copenhagen area)*

At the 25th and 26th floor of Herlev Hospital CAMES covers more than 4000 $m^2$ of specialized simulation and training facilities. In the basement CAMES covers an additional 400 $m^2$ of additional simulation facilities. CAMES Herlev has 16 fully equipped rooms for full scale simulation along with rooms for debriefing, five large classrooms, a training car wreck, a mock-up ambulance and access to a fully equipped ambulance in active service. CAMES has advanced simulators (neonatal, child and adult), less advanced simulators and equipment for training of practical skills.

For additional information:
https://www.regionh.dk/CAMES-english/About-CAMES/contact-cames/Pages/Facilities.aspx

**Course expenses / costs for the 4-day course**

| | Cost per participant in DKK | Cost per participant in EURO |
|---|---|---|
| Instructor salary only | 4963 | 662 |
| Instructor salary incl. tenancy of course facilities, utensils, simulation-mannequin wear and tear | 6500 | 867 |

*DKK: Danish Crowns*

**References**


1 Advanced Life Support Group. Safe Transfer and Retrieval of Patients (STAR): The Practical Approach. 2. edition. BMJ Books 2006.

2 American College of Surgeons. ATLS: Advanced Trauma Life Support for Doctors (Student Course Manual), 8th Edition. 2008.

3 Haig KM, Sutton S, Whittington J. SBAR: a shared mental model for improving communication between clinicians. Jt Comm J Qual Patient Saf. 2006;32:167–75.

4 Sundar E, Sundar S, Pawlowski J, et al. Crew Resource Management and Team Training. Anesthesiol. Clin. 2007;25:283–300.
